# Mid-level Priming by Completion vs. Mosaic Solutions

**DOI:** 10.1177/2041669518820347

**Published:** 2019-04-29

**Authors:** Antonio Peta, Carlo Fantoni, Walter Gerbino

**Affiliations:** Department of Life Sciences, Psychology Unit Gaetano Kanizsa, University of Trieste, Italy

**Keywords:** amodal completion, occlusion, good continuation, contour curvature polarity, symmetry

## Abstract

We report two experiments on the role of mid-level processes in image segmentation and completion. In the primed matching task of Experiment 1, a cue→prime sequence was presented before the imperative stimulus consisting of target shapes with positive versus negative contour curvature polarity and one versus two axes of mirror symmetry. Priming shapes were included in two composite occlusion displays with the same T-junction information and different geometric features supporting a distinct balance between completion and mosaic solutions. A cue, either congruent or incongruent with targets, preceded the presentation of the composite priming display. Matching performance was affected by primes in the expected direction, while cue congruency participated only in a marginally significant three-way interaction, and prime duration had no effect. In Experiment 2, the cue→prime sequence was replaced by a fixation cross to control for the priming effect obtained in Experiment 1. The study confirmed that contour connectability and curvature polarity are effective structural factors capable of competing with symmetry in mid-level image segmentation and completion processes.

## Introduction

Amodal completion refers to the phenomenal presence of object parts lacking the property that characterizes the relevant sensory modality (e.g., color for vision) as well as to processes that allegedly support their formation (Gerbino, 2017). While the importance of amodal phenomena for perceptual science is broadly recognized, underlying processes are more controversial (van Lier & Gerbino, 2015). In vision, perceptual organization goes beyond image segmentation—that is, the unification-segregation of input elements—to include at least the extrapolation of such elements and often their completion in well-formed wholes. In this study, we used a primed matching paradigm to understand how image segmentation and completion are impacted by three mid-level factors: connectability of T-stems, contour curvature polarity (CCP), and mirror symmetry.

Following Fantoni and Gerbino (2013), we use the generic term “connectability” to refer to simplicity of the smooth connection of two contour segments, avoiding specific relatability assumptions (Kellman, 2003; Kellman & Shipley, 1991). We just assume that connectability of symmetric segments monotonically decreases as they depart from collinearity (Fulvio, Singh, & Maloney, 2014).

CCP specifies the local concavity-convexity of the border between adjacent regions. Its role in two-dimensional (2D) shape perception has been reviewed by Bertamini and Wagemans (2013). Strong evidence is available about CCP as a factor in figure/ground articulation (Kanizsa & Gerbino, 1976) and contour interpolation (Fantoni, Bertamini, & Gerbino, 2005). In the present study, CCP covaried with connectability. This is common in natural situations, since occlusion of a rectilinear contour typically generates a locally concave region and a pair of strongly connectable T-stems, while occlusion of a discontinuous contour typically generates a locally convex region and a pair of weakly connectable T-stems.

Mirror symmetry is a prominent factor in 2D shape processing (Bertamini, Silvanto, Norcia, Makin, & Wagemans, 2018). Here, we studied symmetry with respect to one versus two axes and contrasted vertical or horizontal versus 45° oblique orientations, given the well-known dependence of perceived symmetry on orientation (Mach, 1885/1897). In our displays, the tendency toward maximum symmetry favored the mosaic solution, while in previous research it favored the completion solution (van Lier, Leeuwenberg, & van der Helm, 1995).

### Amodal Completion: Phenomena and Processes

Michotte and Burke (1951/1962) introduced the phenomenological notion of *donneé amodal* (amodal datum) to characterize the “invisible” parts that contribute to the experience of object form despite the absence of local color, taken as the modal property of vision.^1^ They discussed two instances of amodal presence: (a) the tunnel effect (i.e., the experience of fluid motion filling in the spatiotemporal interval between disappearance and reappearance of a translating object; Burke, 1952/1962); (b) the experience of solid volume of an opaque three-dimensional object (opposed to the content of the optic array). Both instances involve occlusion: the first by a static screen that covers the central portion of a moving object trajectory and the second by the front surface that makes the rest of the object optically unaccessible. Michotte, Thinès, and Crabbé (1964) extended amodal presence to conditions without occlusion (*à découvert*), exemplified by two phenomena: (a) the curious feeling that the three-dimensional space bounded by a rotating cubic wireframe, though perfectly transparent and immaterial, is captured by the cube and moves with it and (b) the illusory mantle of stereokinetic or stereoscopic lampshades induced by eccentric circular outlines (either rotating or binocularly disparate).

According to the Michotte's school (Wagemans, van Lier, & Scholl, 2006), most instances of visual occlusion, in which an opaque screen interposed between the distal stimulus and the observer leads to the proximal stimulus fragmentation, correspond to perceived objects composed of modal stimulus counterparts and amodal complements. Modal complements not based on local stimulation occur too, but only in the limiting conditions of the Rosenbach (1902) phenomenon (Glynn, 1954/1962), now called “visual phantoms” (Kitaoka, Gyoba, Kawabata, & Sakurai, 2001; Kitaoka, Gyoba, Sakurai, & Kawabata, 2001; Maguire & Brown, 1987; Tynan & Sekuler, 1976). However, occlusion is just the most common ecological event associated with amodal presence, which can also occur without occlusion. In other words, stimulus occlusion is neither necessary nor sufficient for amodal perception, though they are strongly correlated.

According to its proponents (Burke, 1952/1962; Glynn, 1954/1962; Kanizsa, 1954; Kanizsa, 1955/1987; Michotte & Burke, 1951/1962; Michotte et al., 1964), amodal presence is genuinely perceptual (rather than hypothesized or imagined) and depends on the same “complex system of excitations” (Burke, 1952/1962) that determines modal stimulus counterparts. Metaphorically, amodal data are a “bridge” (Burke, 1952/1962; Michotte & Burke, 1951/1962), that is, a construction that unifies otherwise disconnected segments.

Two amodal phenomena are particularly interesting: the Michotte triangle (because of the conflict with the distal stimulus) and the Bregman–Kanizsa effect (because of the conflict with the proximal stimulus).

#### Michotte triangle

**Figure 1.**
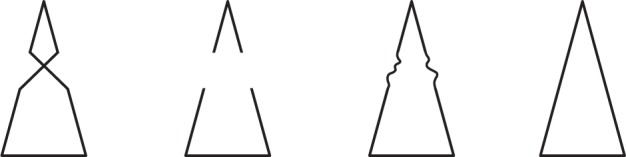
Cover the central part of any pattern (or of all patterns simultaneously). Each will look as a complete isosceles triangle. The left pattern was published by Michotte et al. (1964, Figure 7, p. 19).

In Figure 1, based on Michotte et al. (1964), occlusion of the central region makes each outline pattern appear as a regular isosceles triangle, against the hypothesis that sensory gaps are overcome by observer’s knowledge based upon the immediate memory of the distal object. A real-life large-sized case of amodal completion in contrast with knowledge of the distal stimulus has been recently studied by Ekroll, Mertens, and Wagemans (2018).

#### Bregman–Kanizsa effect

**Figure 2.**
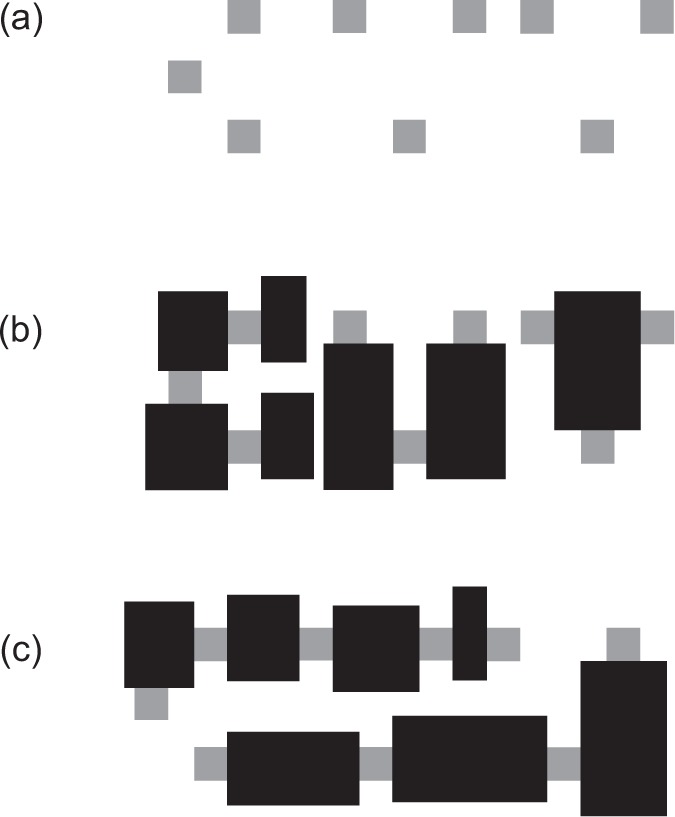
Different objects can emerge in the Bregman–Kanizsa effect. The same gray fragments are available in *a*, *b*, and *c*. But *a* is perceived as a constellation of squares consistent with input regions, while in *b* and *c* (thanks to T-junctions) the gray fragments become the modal parts of alternative wholes, supporting spontaneous letter recognition.

Following Nakayama, Shimojo, and Silverman (1989), this is a common label for the identification gain following amodal completion, compared with a condition in which input fragments are perceived as such. For instance, Kanizsa (1979, Figure 1.1a vs. 1.2b) compared a collection of fragments barely recognizable as pieces of a cubic structure to the same pattern with added T-junctions in which a partially occluded cubic structure becomes salient. Bregman (1981) discussed a pair of pictures where the same fragments are either perceived as such or as the visible parts of easily recognizable amodally completed letters. Figure 2 shows an extreme case of the Bregman–Kanizsa effect in which—like in Kanizsa and Gerbino (1982, p. 177)—amodal completion creates different objects. The same gray squares are present in *a, b*, and *c*, but are perceived as such only in *a,* where they are bounded by closed contours meeting at L-junctions, while they are unified in totally different ways in *b* and *c*, depending on the arrangement of T-junctions and independently of observer’s knowledge of overlearned letters.

The Bregman–Kanizsa effect has been measured using different paradigms (Chen, Liu, Chen, & Fang, 2009; Gerbino, 1981; Johnson & Olshausen, 2005; Murray, Sekuler, & Bennett, 2001). Its strength elucidates why “recognition from partial information,” as used in most computational literature (Bajcsy & Tidhar, 1977; Tang & Kreiman, 2011, 2017), is an inadequate characterization of what happens in perceived occlusion, which benefits from completion processes not activated in the absence of occlusion information.

 Kellman (2000; Carrigan, Palmer, & Kellman, 2016) discussed recognition from partial information as a convenient label for global processes involved in the identification of partially occluded shapes, distinguishable from contour interpolation constrained only by relatability (Kellman, 2003; Kellman & Shipley, 1991), a formalization of the Gestalt principle of good continuation (Wertheimer, 1923). In Experiment 1 by Carrigan et al. (2016), observers localized an amodal contour more precisely when the suggested completion of a partially occluded shape corresponded to a local interpolation than to a globally symmetric supplementation. However, in the absence of a control condition without occluder, the reported difference in precision might be attributed to a general ability to estimate position from spatial cues such as alignment or equidistance from reference points and directions, rather than to amodal completion behind occluders.

Carrigan et al. (2016) interpreted results from their three experiments as evidence that local and global processes involved in the perception of partially occluded 2D shapes are qualitatively different: Only local interpolation would support precise spatial discriminations, while completion based on global processes would be vague and undetermined. Unfortunately, their process dichotomy collapses symmetry and familiarity into a single category (the so-called high-level global processes), while they are heterogeneous factors. The first depends on stimulus-based determinants of local interpolation (contour orientations and positions), whereas the second depends on observer’s specific experience. Furthermore, the degree of determinateness of amodal parts—already discussed by Michotte et al. (1964, p. 18)—may be orthogonal to the type of completion process.

Different dichotomies have been utilized in the amodal completion literature. For instance, phenomenological demonstrations (Kanizsa, 1979, 1985) focused mainly on situations in which perceived shapes are consistent with good continuation of local contours, against expectations based on global regularity. Fantoni and Gerbino (2003) modeled contour interpolation of T-stems as a unification process governed by two basic factors, good continuation and minimal path, with their relative strength modulated by more complex structural factors such as symmetry and CCP. Yun, Hazenberg, and van Lier (2018) contrasted stimulus-based structural factors of different complexity with memory-based knowledge. Further studies based on a dichotomic approach are reviewed in the next section on objective paradigms.

Another dichotomy refers to the distinction between amodal continuation and amodal completion (Anderson, 2007; Gillam, 2003; Minguzzi, 1987). Amodal completion would lie at the interface between perception and cognition in the sense of including both perceptual (amodal continuation) and cognitive (recognition from partial information) components, which in most daily occurrences converge (Kanizsa, 1985). Few (often artefactual) cases of divergence do exist in which the perceptual component prevails, leaving observers surprised by the content of their own phenomenal experience. A paradigmatic effect, in this respect, is the “horse illusion” (Kanizsa, 1970, 1979) in which the front and back parts of two horses are unified into an unlikely long horse, against veridicality. The effect works also for scooters (Kanizsa & Gerbino, 1982) and fruits (Hazenberg & van Lier, 2016).

In principle, the amodal continuation of T-stems could explain both the Michotte triangle and the Bregman–Kanizsa effect, even when the perceived shape of the occluded portion is quite undeterminate. However, other structural factors can affect amodal continuation and therefore amodal completion. They are more complex than good continuation of T-stems (the most elementary factor at the contour level), but qualitatively different from global regularity and familiarity, which refer to visual order and memory-based expectations, respectively.

### Objective Paradigms

Functional effects of amodal completion have been evaluated using objective paradigms such as shape matching, shape discrimination, primed matching, visual search, and others (see reviews by Sekuler & Murray, 2001; Yun et al., 2018). Such studies contributed operational definitions of amodal completion, by measuring its facilitatory or inhibitory effects on the observers’ performance. In the following review, we selected the literature directly relevant to this study, on the basis of two criteria: the paradigm (unprimed/primed shape matching/discrimination) and the focus on structural factors of form organization.^2^

Gerbino (1981) utilized a partial report procedure and found that complete block letters were better discriminated from truncated than retinally identical partially occluded letters (despite the lack of partial-over-whole-report superiority, the hallmark of iconic memory). In his study, amodal completion increased the perceptual similarity between partially occluded and intact letters, inhibiting discrimination with respect to a control condition in which the same letter fragments were available in the absence of occlusion information. Gerbino and Salmaso (1987) used a category matching task and found that complete targets were matched faster to partially occluded than (retinally identical) truncated comparison shapes; by increasing the perceptual similarity between partially occluded and intact polygons, amodal completion facilitated the category match. Their results were corroborated by Shore and Enns (1997), whose parametric study revealed an important monotonic effect of the proportion of occluded area: Amodally completed shapes were equivalent to complete shapes only when such proportion was small, while the time required to match or identify shapes increased as a direct function of the amount of occlusion. Shore and Enns (1997) measured both facilitatory and inhibitory effects of amodal completion: On the one hand, the complete-completed match was facilitated with respect to the complete-truncated match, as in other objective implementations of the Bregman–Kanizsa effect (Bruno & Gerbino, 1987; Gerbino, 1989; Gerbino & Salmaso, 1987); on the other hand, when instructions required observers to adopt a proximal mode of perception, the classification of partially occluded shapes as incomplete suffered from a “perceptual intrusion” due to response inhibition by amodal completion (a Stroop-like effect dependent on the obligatory nature of form integration).

Following Palmer and Sekuler (1988; Sekuler & Palmer, 1992), several studies utilized a primed matching paradigm including a variable-duration prime (complete, amodally completed, or truncated) presented before complete or truncated target pairs, often with the goal of supporting a two-stage model in which the completion solution becomes dominant only after an early mosaic stage. This model has also been tested in a shape discrimination task (Murray et al., 2001). Apart from issues related to the time course of amodal completion, the primed matching paradigm has been used to evaluate the relative strength of local versus global factors.

Sekuler, Palmer, and Flynn (1994) provided evidence that global symmetry can prevail over local good continuation: After discounting a prime-independent symmetry superiority, they found that the effect of a partially occluded prime was more similar to the one by a high-symmetry prime (inconsistent with good continuation) than by a low-symmetry prime (consistent with good continuation).

Van Lier et al. (1995) studied priming as the result of a competition between local completion, global completion, and mosaic solutions. Differently from Sekuler et al. (1994), who considered global completions involving convex protrusions, they considered global completions involving concavities, while local completions were convex in both studies. Both Sekuler et al. (1994) and van Lier et al. (1995) labeled as global the solution with maximum symmetry, while the local solution was, typically, not asymmetric but less symmetric (involving one axis of symmetry instead of two or three). According to van Lier et al. (1995), occlusion configurations can evoke both local and global completions, consistently with similar conclusions by other authors about the parallel context-dependent instantiation of mosaic and completion solutions (Bruno, Bertamini, & Domini, 1997; Bruno & Gerbino, 1987; Gerbino, 1989; Gerbino & Salmaso, 1987; Rauschenberger, Peterson, Mosca, & Bruno, 2004).

Plomp and van Leeuwen (2006) introduced a complex two-prime paradigm, to evaluate the possible effect of a single prime on a composite prime and, consequently, on target matching. They obtained evidence that single primes can facilitate local completions, global completions, as well as mosaic interpretations of the composite prime, but only when the latter was presented briefly (50 vs. 500 ms).

Emmanouil and Ro (2014) focused on the effect of an unconsciously completed prime on target discrimination. The prime was presented so briefly that its identification—in a control session—was at chance. In their first experiment, observers should discriminate between a full disk and a pacman, after one of four possible primes: disk, pacman, occluded disk, and neutral (misaligned disk portions equivalent to the visible portions of the occluded disk). The pacman prime was combined with an outline rectangle in a complex way: On one side, a pair of T-junctions supported the amodal continuation of the outline rectangle behind the protruding convex portion of the pacman; on the other side, pacman and rectangle contours joined at locally ambiguous fork junctions, while the local concavity of the pacman supported its amodal continuation behind the adjacent rectangle portion. Not surprisingly, the pacman prime did not produce the expected facilitation on response speed for pacman targets, with respect to the neutral prime. However, the overall response speed pattern was consistent with priming by unconsciously completed shapes.

## Experiment 1

Consistently with the basic assumption that amodally completed primes can facilitate the processing of similar shapes, the main goal of Experiment 1 was to test whether composite priming (P) displays that favor either completion or mosaic solutions can affect the simultaneous matching of 2D targets (T) corresponding to such solutions.

**Figure 3.**
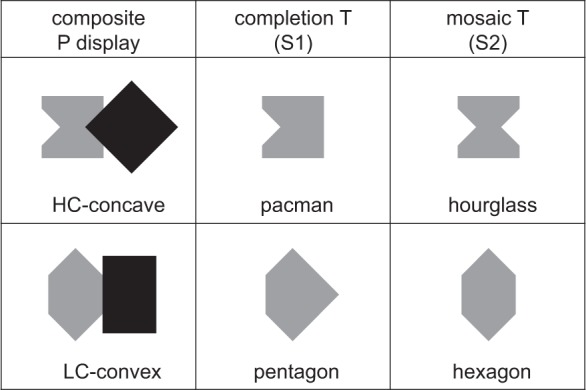
Composite priming (P) displays and targets (T) in Experiment 1. Gray regions of P displays differed with respect to contour connectability and CCP. Amodal completion was equally supported by T-junctions and (a) strengthened by high connectability (HC) and mosaic concavity in the hourglass + diamond display or (b) weakened by low connectability (LC) and mosaic convexity in the hexagon + rectangle display. Targets were labeled “completion T” and “mosaic T” with reference to competing solutions of the segmentation of the respective P display. Completion Ts had one symmetry axis (S1), while mosaic Ts had two (S2).

Figure 3 shows the two P displays and the four T shapes (two for each P display) used in Experiment 1. The composite P displays were selected to keep under control several structural factors known to affect the contour interpolation path and the strength of amodal completion of 2D shapes. In both cases, the prime (i.e., the stimulus expected to affect target matching) was the gray region on the left of the black region intended as an occluding surface. Hourglass and hexagon regions were derived from a unit square by means of the truncation of two right-angle isosceles triangles (hypotenuse = *ax*; with *a* = [$\sqrt{2}$ / (1 + $\sqrt{2}$)], and *x* = side of the unit square). Consequently, all hexagon sides had an equal length, the two primes had an equal area, and the hypothetical completion regions bounded by straight T-stem extrapolations had an equal area too. In both P displays, the good-continuation completion would correspond to a nominal surface increment of about 9%. The amount of support ratio at the contour level—that is, the ratio of input length to total length (always according to good continuation only)—differed for hourglass-to-pacman and hexagon-to-pentagon cases (0.41 vs. 0.59, respectively). This difference was intended to compensate, at least partially, the weaker tendency to completion in the hexagon + rectangle display expected on the basis of the main factors involved in the segmentation of the two composite P displays, which were the following:

- Local T-junction information: Both composite P displays contained two 45° T-junctions that, taken separately, conveyed the same information about the amodal continuation of a gray surface behind a black occluder, on top of a white background. Hence, with respect to *local T-junction information*, the two composite P displays were equivalent.
- Connectability of T-stems: This is a higher level property compared with the mere presence and number of independent T-junctions. In the hourglass + diamond display, the interpolation was collinear, leading to a high connectability (HC) condition; while in the hexagon + rectangle display, extrapolations of the two T-stems met at a 90° angle, leading to a low connectability (LC) condition, perceptually solved as an amodal rounded angle (Fantoni & Gerbino, 2003; Gerbino, 2017; Gerbino & Fantoni, 2006). Hence, with respect to *connectability of T-stems*, the amodal continuation of gray-on-white borders behind the black occluder was stronger in the hourglass + diamond display than in the hexagon + rectangle display.
- CCP: The priming region was concave in the hourglass + diamond display and convex in the hexagon + rectangle display. Hence, with respect to *CCP*, concavity avoidance (i.e., the tendency to maximize convexity and eliminate concavities) supported the completion solution in the hourglass + diamond display, while it was neutral in the hexagon + rectangle display, where completion and mosaic solutions were both convex.
- Mirror symmetry: In both cases, amodal continuation behind the black occluder entailed the loss of mirror symmetry with respect to the vertical axis and only allowed for the maintenance of symmetry relative to the horizontal axis. Hence, with respect to *mirror symmetry*, the two primes were equivalent.

To summarize, the composite P displays differed with respect to connectability and CCP*,* while they were equivalent with respect to local T-junction information and mirror symmetry. In one case, amodal completion of the hourglass into a pacman behind an occluding diamond (by elimination of one of the two local gulfs) entailed a loss of regularity but a gain in convexity and contour smoothness; in the other case, amodal completion of a hexagon into a pentagon behind a rectangle entailed a loss of regularity but no gain in convexity and only a partial gain in contour smoothness (one discontinuity in the pentagonal contour instead of two in the hexagonal contour). Hence, the relative prevalence of the completion over mosaic solution was expected to be higher in the HC-concave display than in the LC-convex display.

Participants were required to perform a *same-different* discrimination of 2D target shapes corresponding to completion or mosaic solutions of composite P displays. Completion targets (pacman and pentagon) were polygons with only one axis of mirror symmetry (S1), while mosaic targets (hourglass and hexagon) were polygons with two axes of mirror symmetry (S2), as indicated in the upper row of Figure 3. Like in Sekuler et al. (1994), though with other shapes, target symmetry covaried with the segmentation solution: In both studies, the low symmetry solution corresponded to completion according to good continuation, whereas the high symmetry solution corresponded to a completion with a convex protrusion in Sekuler et al. (1994) and to the mosaic solution in the present experiment.

**Figure 4.**
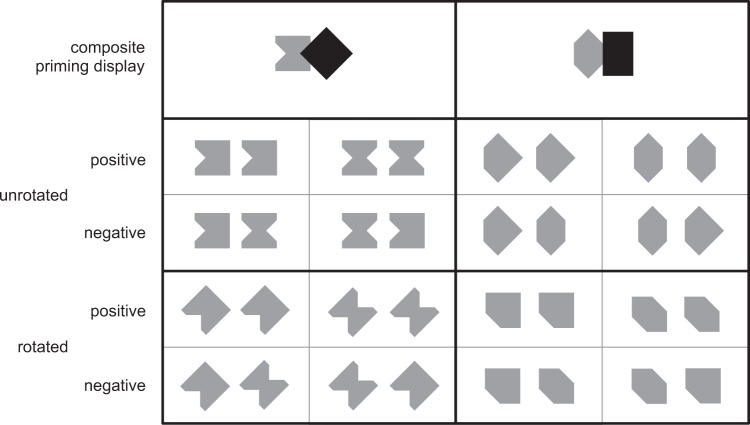
Composite P displays and T-pairs used in positive and negative trials. The 8 T-pairs presented after the hourglass + diamond display are shown on the left; the 8 T-pairs presented after the hexagon + rectangle display on the right. Note that each subset of target pairs (concave on the left vs. convex on the right) was preceded by it own prime (HC-concave vs. LC-convex, respectively).

Figure 4 shows the whole set of composite P displays and T-pairs used in positive and negative trials of our simultaneous matching task. The HC-concave hourglass + diamond P display was followed by one out of four fully balanced T-pairs: two pacmen or two hourglasses in positive trials (correct response *same*) and one pacman and one hourglass in negative trials (correct response *different*). The LC-convex hexagon + rectangle P display was followed by one out of four fully balanced T pairs: two pentagons or two hexagons in positive trials and one pacman and one hourglass in negative trials.

Composite P displays were always presented in the orientation shown in the upper row of Figure 4, while the two targets were presented either unrotated (middle row) or rotated 45° counterclockwise (bottom row).

In general, we expected to find a symmetry superiority for matching hourglasses over pacmen and hexagons over pentagons (i.e., a relative difference between speed and accuracy of *same-different* responses to S2- and S1-targets), as a simple consequence of higher shape regularity, independent of priming (Bertamini et al., 2018; Garner, 1974; Sekuler et al., 1994).

Given the experimental conditions, priming was conceptualized as a prime-dependent modulation of symmetry superiority. Since the HC-concave P display (because of the higher relative prevalence of completion over mosaic) should act against symmetry and facilitate pacmen more than hourglasses, while the LC-convex P display (because of the lower relative prevalence of completion over mosaic) should act consistently with symmetry and facilitate hexagons more than pentagons; in the unrotated condition, the amount of symmetry superiority was expected to be smaller for hourglasses over pacmen than hexagons over pentagons.

In the rotated condition, if priming does not generalize to target shapes presented in a conflicting orientation because of a loss of prime-target perceived similarity, the difference between symmetry superiorities in the two priming conditions (HC-concave vs. LC-convex displays) should be reduced or absent. Collinearity with the cardinal axes of visual space made different target features salient in unrotated versus rotated orientations: for example, the long sides of the pentagon, which were oblique in the unrotated-target condition, became vertical or horizontal in the rotated-target condition.

The design of Experiment 1 included two further manipulations, aimed at evaluating whether the shape of a preprime cue and prime duration could possibly affect prime segmentation, as revealed by the amount of symmetry superiority in the simultaneous matching task. As found by Plomp and van Leeuwen (2006), a single shape, briefly presented before the composite P display, might cue its segmentation. For each P display, the completion cue was identical to the completion T (S1) and the mosaic cue to the mosaic T (S2). The second effect was related to the duration of the P display. Following Plomp and van Leeuwen (2006), we selected two durations (50 and 500 ms) to replicate the possible prevalence of mosaic over completion solutions at short exposures.

### Methods

#### Participants and ethical approval

Twenty-five undergraduates (17 females; mean age 23.2 years) voluntarily took part in the study. All had normal or corrected-to-normal vision. Methods and procedures were approved by the Ethical Committee of the University of Trieste (n. 84c/2017), and verbal informed consent at recruitment was obtained by the experimenter (the first author).

We decided to rely on a sample of 25 participants following the results of a sensitivity analysis (G*Power 3.1; Faul, Erdfelder, Lang, & Buchner, 2007) focused on an interaction in a repeated measure two-way design representative of the expected priming effect: that is, a difference between symmetry superiorities for concave versus convex shapes larger in the unrotated than rotated condition. Given a 0.5 correlation among repeated measures, α err prob = 0.05, power (1 − β err prob) = 0.8, and *N* = 25, the minimum detectable effect size corresponded to Cohen’s *f* = 0.292 ($\eta_{\text{p}}^{2}$ = 0.078).

#### Apparatus, stimuli, and procedure

**Figure 5.**
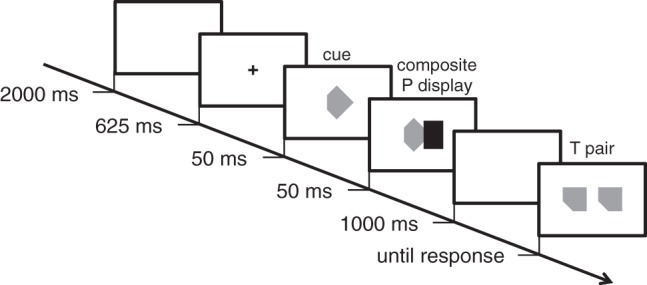
Example of a positive trial in Experiment 1, with rotated convex S1-targets presented after the LC-convex P display preceded by a completion cue (hence, geometrically congruent with both targets). Timing was the same in all trials, with the only exception of composite P display duration (either 50 or 500 ms).

An Intel® Core™ i7 laptop running *SuperLab 4.0* (www.cedrus.com) was used for presenting stimuli on a 15.6-in. color screen monitor and collecting responses from the computer keyboard. Participants were seated approximately 57 cm from the computer screen in a dimly illuminated room. Cue, prime, and target regions appeared mid gray (28 cd/m^2^), while diamond and rectangle of composite P displays appeared black (5 cd/m^2^) and the background white (85 cd/m^2^). A trial is exemplified in Figure 5. The side of the unit square from which cue, prime, and target regions were cut out measured 70 mm on screen. Target pairs subtended a maximum extent of 20° horizontally and 10° vertically.

After a practice block (16 trials), participants completed two experimental blocks (128 trials each), separated by short breaks. Instructions required to press quickly and accurately one of two keys to signal whether the two targets were *same or different*. Feedback regarding accuracy was given during the practice block but not during experimental blocks. Half participants were asked to use their dominant hand for the *same* key and the other half for the *different* key.

The whole experimental set included 256 trials, presented in a fully randomized sequence different for every participant: 128 positive trials—4 repetitions × Rotation (unrotated, rotated) × CCP (concave, convex) × Symmetry (1, 2) × Cue (congruent, incongruent) × Exposure (50 ms, 500 ms)—and 128 negative trials—8 repetitions × Rotation × CCP × Cue × Exposure. To familiarize participants with stimuli and task, the practice block included eight positive and eight negative trials, randomly extracted from the respective subsets, anew for each participant. A session lasted about 30 min. The experimenter (AP) delivered instructions and sat in the experimental room during the whole session, monitoring task execution without looking at the screen where stimuli were displayed.

#### Data analysis

Given the small number of repetitions in each cell of the five-factor within-subject design underlying the set of positive trials, data were analyzed in two steps.

1. First, we ran an LME analysis of speed of correct responses in positive trials (Hits) on the whole within-subject design, with five dichotomous factors—Rotation (unrotated, rotated), CCP (concave, convex), Symmetry (S1, S2), Cue (congruent, incongruent), Exposure (50 ms, 500 ms)—and Subject as a random factor. A total of 2,925 Hit speed values entered the LME analysis, out of the total of 3,200 positive trials resulting from the product of 25 Participants × 128 Positive Trials. Response speed was computed as the inverse of response time (i.e., 1/RT; with RT in seconds). As discussed by Whelan (2008), such a transformation tends to normalize the asymmetric distribution of raw RTs. In our paradigm, response time included observation time, given that target presentation was terminated by response keypress. We did not compute *d*′ values for the five-factor design, given that *p*(Hit) values would be based on a too small number of trials (max = 4) and for two participants there were no correct responses in some cells of the five-factor design.
2. The outcome of the first step of data analysis allowed us to disregard two factors (Cue and Exposure) and focus on the main goal of the experiment; that is, on the effect of the composite P display on simultaneous matching. This was done by computing individual values for speed, *d*′ and *k* = *d′*/√RT (with RT in *s*) for every participant in each condition of the Rotation × CCP × Symmetry design. For the rationale behind this synthetic index, see Fantoni, Gerbino, and Kellman (2008). Individual speed estimates were computed by taking the trimean (Tukey, 1977) of 1/RT values for hits, out of 16 positive trials per condition. Individual *d′* values were obtained from *p*(Hit) out of 16 positive trials and *p*(FA) out of 32 negative trials, applying the conventional 1/(2 *n*) correction to extreme proportions. To highlight the expected priming effect, we ran a contrast analysis of symmetry superiority using the relative difference (per cent Michelson contrast) of each performance index as a score: *M* = 100 × [(S2 − S1) / (S2 + S1)]. To test the priming effect, we ran a planned contrast between (convex–concave) symmetry superiority differences in unrotated (*D*_u_) versus rotated (*D*_r_) conditions:
  $$\begin{array}{c} D_{\text{u}}=[M_{\text{convex}}-M_{\text{concave}}{]}_{\text{unrotated}};\\ D_{\text{r}}=[M_{\text{convex}}-M_{\text{concave}}{]}_{\text{rotated}}. \end{array}$$

Differences between means and against zero were tested using a two-tailed Student’s *t* test. To evaluate all possible effects in the three-way design, we also ran separate three-way analyses of variance (ANOVAs) on speed, *d′* and *k* data. For simplicity, only *k*-based results are reported in the main text, while speed- and *d′*-based results are reported in the Appendix.

### Results

#### LME analysis of response speed in the five-factor design

We used R packages to perform an LME analysis with Rotation, CCP, Symmetry, Cue, and Exposure as fixed effects and Subjects as random factor using the Satterthwaite approximation for degrees of freedom. The outcome was clear-cut. Neither the main effects of Cue and Exposure nor interactions involving these two factors were significant,^3^ with only the Rotation × CCP × Cue interaction just above significance, *F*(1, 2869.5) = 3.84, *p* = .0501, $\eta_{\text{p}}^{2}$ = 0.001. As expected, response speed was strongly influenced by target Symmetry, independent of other factors, *F*(1, 2872.6) = 212.27, *p* < .001, $\eta_{\text{p}}^{2}$ = 0.069. The main effect of CCP and all two-way interactions involving Rotation, CCP, and Symmetry factors were significant, while the main effect of Rotation was above significance.^4^ More importantly—being consistent with the expected priming effect—also the Rotation × CCP × Symmetry interaction was significant, *F*(1, 2869.7) = 4.28, *p* < .05, $\eta_{\text{p}}^{2}$ = 0.001.

**Figure 6.**
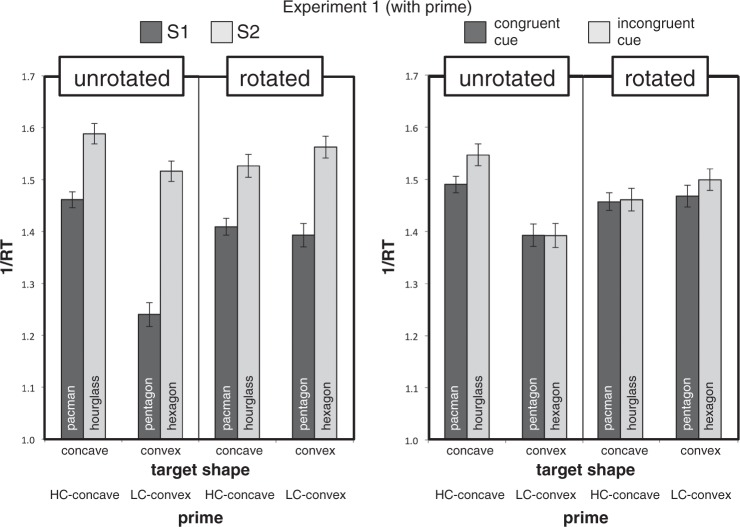
Mean values of 1/RT (± 1 *SEM*) for correct same responses (Hits) in Experiment 1. The left graph shows data for the Rotation × CCP × Symmetry design; the right graph for the Rotation × CCP × Cue design.

The left graph in Figure 6 shows mean response speeds in the reduced Rotation × CCP × Symmetry design. The two patterns of four means in unrotated versus rotated conditions differed in the expected direction. Response speed in the rotated baseline condition was affected only by Symmetry, while in the unrotated condition, the symmetry superiority for concave targets (hourglass over pacman) was smaller than the one for convex targets (hexagon over pentagon).

To understand the meaning of the marginally significant Rotation × CCP × Cue interaction (right graph of Figure 6), we tested the robustness of the strong Rotation × CCP interaction across the two levels of Cue congruency. We ran two separate analyses of the four-way design with Rotation, CCP, Symmetry, and Exposure as fixed effects and Subjects as random factor, one for the congruent Cue condition and the other for the incongruent Cue condition. In both cases, the Rotation × CCP interaction remained highly significant, congruent Cue: *F*(1, 1612.7) = 12.38, *p* < .001, $\eta_{\text{p}}^{2}$ = 0.008; incongruent Cue: *F*(1, 1236.3) = 33.15, *p* < .001, $\eta_{\text{p}}^{2}$ = 0.026, with a similar pattern of the four means, as shown in the right graph of Figure 6. The LME-estimated response speed gain due to concavity was indeed significant for unrotated but not rotated displays, in both incongruent (unrotated = 0.1192 ms, χ^2 ^= 27.004, *p* < .001; rotated = 0.0074 ms, χ^2 ^= 0.111, *p* = .739) and congruent (unrotated = − 0.165 ms, χ^2 ^= 43.720, *p* < .001; rotated = − 0.0366 ms, χ^2 ^= 2.221, *p* = .136) Cue conditions. Such results indicate that the Rotation × CCP interaction was robust across cue variations. However, cue congruency accounted for a residual small modulation of the crossover between Rotation × CCP conditions, with response speed to concave displays always larger for unrotated than rotated displays but significantly in the incongruent Cue condition (LME-estimated difference = −0.0786, χ^2 ^= 10.365, *p* = .0013), while not in the congruent Cue condition (LME-estimated difference = −0.0340, χ^2 ^= 2.819, *p* = .0932). Conversely, response speed for convex displays was significantly larger for rotated than unrotated displays in both Cue conditions (LME-estimated difference for the congruent Cue = 0.1232, χ^2 ^= 24.035, *p* < .001; LME-estimated difference for the incongruent Cue = 0.0778, χ^2 ^= 10.105, *p* = .0015).

#### Test of the priming effect within the reduced three-way design

Given the outcome of the LME analysis, we computed individual speed trimeans, *d′* and *k* values for the Rotation × CCP × Symmetry design. The positive correlation between the speed and *d′* group means for the eight conditions of the three-way design was significant, *r* = .944; *t*(6) = 7.017, *p* < .001. The individual correlation was negative for only three participants out of 25. The prevalence of a positive speed-*d′* correlation supported the choice of *k* as a valid index of overall matching performance.

**Figure 7.**
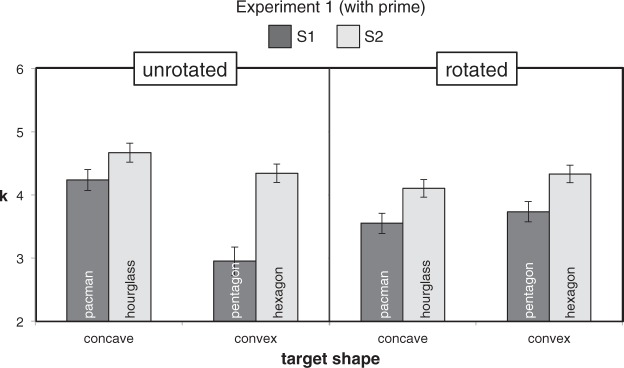
Mean *k* values (± 1 *SEM*) in the within-subject Rotation × CCP × Symmetry design of Experiment 1 after pooling data from different cue and prime duration trials.

**Figure 8.**
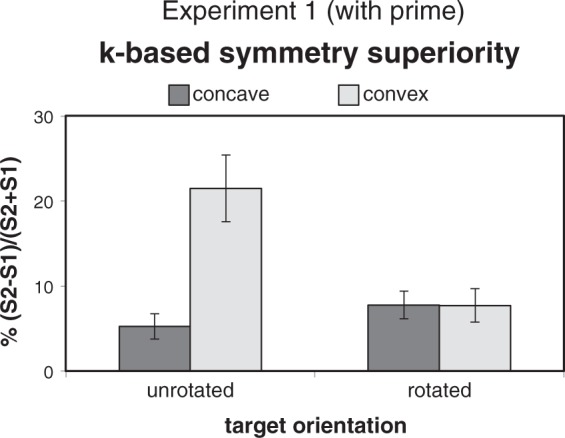
Mean values (± 1 *SEM*) of *k*-based symmetry superiority in Experiment 1. Symmetry superiority was larger than zero in each of the four conditions of the reduced Rotation × CCP design. The expected priming effect consisted in the presence of a larger symmetry superiority for the convex (hexagons over pentagons) than concave (hourglasses over pacmen) pair in the unrotated condition not obtained in the rotated condition.

Mean *k* values for the three-way design are shown in Figure 7 and the derived symmetry superiority M values for the Rotation × CCP design are shown in Figure 8. The expected priming effect was supported by the significance of the planned contrast between the *k*-based symmetry superiority difference for unrotated (*D*_u_) versus rotated (*D*_r_) target pairs, 16.25 versus −0.07%; *t*(24) = 3.90, *p* < .001, Hedges’s *g* = 0.96.

Consistently with the main effect of Symmetry in the LME analysis, all four *k*-based symmetry superiority means in Figure 8 were larger than zero, *t*(24) > 3.53, *p* < .002, Cohen’s *d* > 0.71. A two-way ANOVA showed the significance of all effects, Rotation: *F*(1, 24) = 7.75, *p* < .02, $\eta_{\text{p}}^{2}$ = 0.244; CCP: *F*(1, 24) = 10.80, *p* < .005, $\eta_{\text{p}}^{2}$ = 0.310; Rotation × CCP interaction: *F*(1, 24) = 15.20, *p* < .001, $\eta_{\text{p}}^{2}$ = 0.388. In the unrotated condition, symmetry superiority was larger for convex (hexagons over pentagons) than concave (hourglasses over pacmen) targets, 21.50 versus 5.25%: *t*(24) = 4.010, *p* < .001, Hedges’s *g* = 1.03. Symmetry superiority for convex targets was significantly larger in the unrotated versus rotated condition, 21.50 versus 7.72%: *t*(24) = 3.874, *p* < .002, Hedges’s *g* = 0.81, while the one for concave targets was not significantly smaller in the unrotated versus rotated condition, 5.25 versus 7.79%: *t*(24) = 1.395, *p* = .176. The two means in the rotated condition did not differ (7.79 vs. 7.72%: *t* < 1).

In the earlier analyses the basic score was the relative difference between performance on the two levels of Symmetry. To fully explore the data pattern, we also ran a three-way ANOVA on absolute *k* values (Figure 7). Apart from the main effect of Rotation, *F*(1, 24) = 1.98, *p* = .173, all effects were significant, CCP: *F*(1, 24) = 8.12, *p* < .01, $\eta_{\text{p}}^{2}$ = 0.026; Symmetry: *F*(1, 24) = 57.30, *p* < .001; $\eta_{\text{p}}^{2}$ = 0.705; Rotation × CCP: *F*(1, 24) = 24.90, *p* < .001, $\eta_{\text{p}}^{2}$ = 0.509; Rotation × Symmetry: *F*(1, 24) = 7.76, *p* < .02, $\eta_{\text{p}}^{2}$ = 0.244; CCP × Symmetry: *F*(1, 24) = 10.10, *p* < .005, $\eta_{\text{p}}^{2}$ = 0.296; Rotation × CCP × Symmetry: *F*(1, 24) = 13.60, *p* < .002, $\eta_{\text{p}}^{2}$ = 0.362.

### Discussion

Experiment 1 confirmed the importance of symmetry in the simultaneous matching of 2D shapes: Independent of various experimental manipulations, *same* responses were faster and more accurate for S2- than S1-targets, supporting the adoption of symmetry superiority as an appropriate dependent measure.

Results were quite informative about the effect of the composite prime display (the main goal of our study), considering that S1- and S2-targets corresponded to completion and mosaic solutions of the composite P display segmentation, the first favored in the hourglass + diamond display (concave condition) and the second in the hexagon + rectangle display (convex condition), respectively. The difference between convex and concave conditions was dependent on target rotation. As expected, in the unrotated condition (targets oriented as the prime), the geometric mid-level features of the prime (HC-concave vs. LC-convex) affected the amount of symmetry superiority, which turned out to be weaker for hourglass over pacman than hexagon over pentagon. Symmetry superiority for convex versus concave targets did not differ in the rotated condition, suggesting that priming—at least in the conditions of our experiment—was not orientation invariant. We interpret this lack of orientation invariance as a consequence of a loss of perceptual prime-target similarity reducible to Mach’s square/diamond phenomenon (i.e., to the dependence of perceived form on collinearity of its structural elements with the cardinal axes of visual space).

However, this interpretation poses a problem for the evaluation of the hypothetical components of the overall priming effect measured in the unrotated condition, assuming that it should, depending on the P display, either inhibit maximum symmetry (when the P display was HC-concave) or facilitate it (when the P display was LC-convex). If the rotated condition was simply a no-priming condition (with symmetry superiority unaffected by rotation *per se*), then both components of priming should be significant: the hexagon over pentagon symmetry superiority, attributable to the LC-convex P display, should be larger in the unrotated than rotated condition, as found; but on the same grounds, the hourglass over pacman symmetry superiority, attributable to the HC-concave P display, should be smaller in the unrotated than rotated condition. In Experiment 1, this second difference, though in the expected direction, did not reach significance.

We attributed such pattern of results to a possible intrinsic priming-independent effect of rotation on symmetry superiority, affecting both target pairs (hourglass over pacman and hexagon over pentagon). In other words, the relatively low symmetry superiority in the rotated condition could simply reflect the reduced salience of mirror symmetry along oblique axes, making this condition only a partially appropriate control for simultaneous matching in the unrotated condition. Experiment 2, in which the same targets were not preceded by a prime, could provide evidence relevant to this point.

From the statistical point of view, Experiment 1 provided us with inconclusive evidence about cue congruency and prime duration. However, specific interpretations can be suggested for the lack of significance in the two cases.

As regards cue congruency, the analysis of response speed for the full five-way design indicates that the presentation of the 50-ms cue before the prime might weakly modulate the performance superiority for concave over convex unrotated targets (larger in the incongruent than congruent cue condition, as shown in the right graph of Figure 6). This marginally significant effect of cue congruency, if combined with evidence of prime effectiveness, is consistent with the dominance of autochthonous structural factors over immediate memory in image segmentation, which is the meaning of the Michotte triangle demonstration (Figure 1).

As regards prime duration, the lack of difference between 50- and 500-ms primes is attributable to the long blank ISI between prime offset and target pair onset (1,000 ms). Even in the context of a two-stage model of amodal completion—criticized by Bruno et al. (1997) and Rauschenberger et al. (2004), among others—shape processing cannot be blocked at the hypothetical mosaic stage if prime availability is not terminated by backward masking. Plomp and van Leeuwen (2006) did find a differential effect of composite prime duration (50 vs. 500 ms), but their paradigm—though lacking an after-prime mask—included more levels of congruency between targets and preceding shapes, making a direct comparison with our experiment difficult.

## Experiment 2

To evaluate whether symmetry superiorities found in Experiment 1 (Figure 8) could be partially attributed to matching difficulty intrinsic to various targets, we ran Experiment 2, which paralleled Experiment 1 in all respects, except for the absence of the cue→prime sequence and the reduction of the intertrial interval.

As a consequence of prime removal, we expected the disappearance of the significant Rotation × CCP interaction found in Experiment 1 (Figure 8). A replication of such interaction would be incompatible with our interpretation of Experiment 1 and, in particular, would undermine the attribution of the (convex–concave) symmetry superiority difference in the unrotated condition to priming.

### Methods

#### Participants

Twenty volunteers (eight females; mean age 27.0 years) took part in the study. For details of the ethical approval, see Experiment 1. All participants had normal or corrected-to-normal vision.

As for Experiment 1, we decided to rely on a sample of 20 participants following the results of a sensitivity analysis (G*Power 3.1; Faul et al., 2007) focused on an interaction in a repeated measure two-way design. Given a 0.5 correlation among repeated measures, α err prob = 0.05, power (1 − β err prob) = 0.8, and *N* = 20, the minimum detectable effect size corresponded to Cohen’s *f* = 0.331 ($\eta_{\text{p}}^{2}$ = 0.099).

#### Apparatus, stimuli, and procedure

Apparatus and procedure were identical to those in Experiment 1. As regards stimuli: The cue→prime sequence was substituted by a central fixation cross lasting either 100 or 550 ms, to parallel the within-trial timing of Experiment 1 in which the cue lasted 50 ms and the prime either 50 or 500 ms; to compensate for stimulus simplification, the duration of the blank intertrial interval was 1,000 ms (instead of 2,000 ms in Experiment 1).

Hence, the 128 positive trials included 16 repetitions for each combination of the Rotation × CCP × Symmetry design, while the 128 negative trials included 32 repetitions of the Rotation × CCP design.

#### Data analysis

As in Experiment 1, we computed speed, *d*′ and *k* values. Only the output of *k*-based analyses is reported in the main text, while speed- and *d*′-based analyses are reported in the Appendix.

### Results

The speed-*d′* correlation between the two sets of eight group means from the three-way design was positive but not significant, *r* = .520; *t*(6) = 1.489, *p* = .187. The individual correlation was negative for eight participants out of 20. Likely, the larger proportion of participants with a speed-*d′* tradeoff in Experiment 2 (χ^2 ^= 4.72, *p* < .05) contributed to the substantial decrease in the positive correlation between group means, with respect to Experiment 1. Therefore, conclusions from *k*-based analyses should be integrated by an evaluation of the output of separate analyses of speed- and *d′-*based analyses reported in the Appendix.

**Figure 9.**
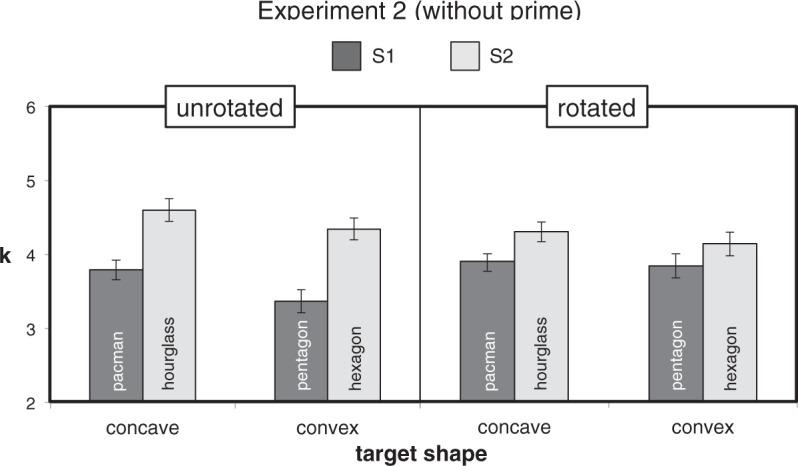
Mean *k* values (±1 *SEM*) in the within-subjects Rotation × CCP × Symmetry design of Experiment 2. Evidence of the overall effect of priming was not replicated (see Figure 7 for comparison).

Mean *k* values for the Rotation × CCP × Symmetry design of Experiment 2 are shown in Figure 9. As in Experiment 1, the main effects of Symmetry, *F*(1,19) = 17.10, *p* < .001, $\eta_{\text{p}}^{2}$ = 0.474, and CCP, *F*(1,19) = 7.64, *p* < .02, $\eta_{\text{p}}^{2}$ = 0.287, were significant, while the main effect of Rotation was not (*F* < 1). But differently from Experiment 1, only the Rotation × Symmetry interaction was significant, *F*(1,19) = 32.40, *p* < .001, $\eta_{\text{p}}^{2}$ = 0.630; Rotation × CCP: *F*(1,19) = 2.92, *p* = .104; CCP × Symmetry: *F* < 1; Rotation × CCP × Symmetry: *F*(1,19) = 1.47, *p* = .240. The absence of a significant three-way interaction was consistent with the idea that symmetry superiority, within each Rotation condition, was equal for concave and concave targets, contrary to Experiment 1.

**Figure 10.**
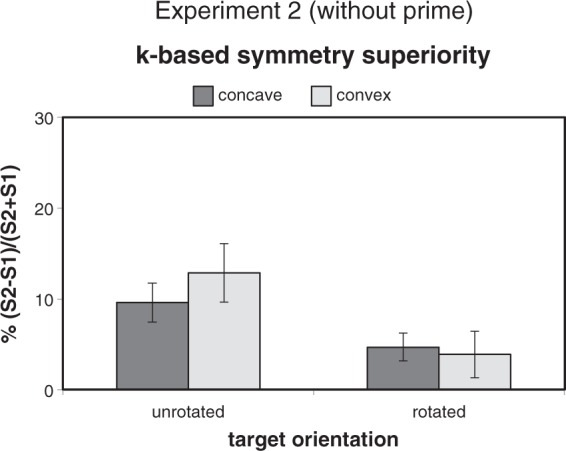
Mean values (±1 *SEM*) of *k*-based symmetry superiority in Experiment 2. Evidence of the overall priming effect found in Experiment 1 was not replicated (see Figure 8 for comparison).

To control for such an interpretation, we also ran a two-way ANOVA on *k*-based symmetry superiority scores (Figure 10). The main effect of Rotation was significant, *F*(1, 19) = 24.30, *p* < .001, $\eta_{\text{p}}^{2}$ = 0.561, while the main effect of CCP (*F* < 1) and the interaction, *F*(1, 19) = 2.01, *p* = .172, were not. Mean symmetry superiorities for concave and convex targets were both significantly larger than zero in the unrotated condition, concave 9.6%: *t*(19) = 4.45, *p* < .001, Cohen’s *d* = 0.996; convex 12.9%: *t*(19) = 3.23, *p* < .001, Cohen’s *d* = 0.89, whereas in the rotated condition, the mean symmetry superiority was larger than zero for concave 4.7%: *t*(19) = 3.11, *p* < .01, Cohen’s *d* = 0.6954; convex 3.9%: *t*(19) = 1.52, *p* = .145.

Symmetry superiority was larger in the unrotated than rotated condition, suggesting that this aspect of the data pattern, found also in Experiment 1, was independent of priming and related to the intrinsic role of orientation on perceived form.

## Conclusions

Taken together, the two experiments provided evidence that the composite priming displays affected the simultaneous matching of 2D polygonal shapes, consistently with mid-level factors favoring either completion (for the hourglass + diamond prime) or mosaic (for the hexagon + rectangle prime) solutions. Within the design of Experiment 1, priming was operationalized as a modulation of symmetry superiority; that is, as a change in the intrinsic relative difficulty of matching hourglasses over pacmen and hexagons over pentagons.

In the unrotated condition of Experiment 1, we obtained a small symmetry superiority for concave targets (hourglasses over pacmen, presented after a composite priming display in which amodal completion was favored by HC and concavity avoidance) and a large symmetry superiority for convex targets (hexagons over pentagons, presented after a composite priming display in which the mosaic solution was favored by LC and convexity). This difference between concave and convex targets was not obtained in the rotated condition of Experiment 1 when priming was ineffective because of the misorientation-dependent loss of prime-target similarity.

Experiment 2 provided another control for the critical role of the prime. In the absence of the prime (Experiment 2), the symmetry superiorities for concave versus convex targets did not differ in either rotation condition (Figure 10), supporting the idea that the difference found in the unrotated condition of Experiment 1 (Figure 8) should be attributed—at least partially, if not totally—to prime presentation. The validity of Experiment 2 as a control for Experiment 1 should be supported by experiments with prime presentation as a design factor, allowing for a direct test of its effect.

The aforementioned conclusion refers to analyses conducted on *k* values and derived scores. However, the analyses of speed and *d ′* data reported in the Appendix revealed an important difference between Experiments 1 and 2. In Experiment 1, where speed and *d ′* measures were highly correlated, evidence of the expected priming effect was found within each data distribution. On the other hand, in Experiment 2, where the positive correlation between the two measures was not significant and a larger proportion of participants exhibited a speed-*d ′* tradeoff, the planned comparison between symmetry superiority differences was significant for *d ′*-based scores but not for speed-based scores, though the direction of the two effects was consistent. Also, this aspect of our data requires further research.

As regards amodal completion processes, our study supports the role of mid-level factors as determinants of the segmentation of 2D composite P displays and of amodal continuation strength, beyond local T-junction information. Results from our two experiments are compatible with the hypothesis that in Experiment 1 the two occlusion displays produced opposite priming effects on the respective targets. The different balance of completion versus mosaic solutions produced by the conjoint action of connectability and CCP modulated the intrinsic symmetry superiority found in matching targets with versus without a vertical axis of symmetry.

Establishing the relative contribution of connectability and CCP to the segmentation and amodal completion of occlusion patterns was beyond the scope of this study. As a first step, we were interested in demonstrating the relevance of the combination of such factors, often associated in occlusion optics, while keeping T-junction information constant.

In Experiment 1 we tried to evaluate two other possible effects, besides priming by the composite priming display presented immediately before the imperative stimulus. One effect should depend on cueing the segmentation of the composite P display by a shape corresponding to either a completion or mosaic solution (hence, identical to either S1- or S2-targets in the matching task). The other effect should depend on prime duration, under the assumption that completion prevails over the mosaic solution as the exposure duration increases.

Neither cue congruency by itself nor its interaction with target rotation had any effect of matching speed. The marginal interaction between cue congruency, target rotation, and CCP obtained in the five-way ANOVA on response speeds in Experiment 1—suggesting the possibility that the cue is effective when unrotated targets are concave, but not convex—requires further research. However, the weak or null effect of cue congruency is open to at least two interpretations. Suppose that the cue affects the segmentation of the composite prime but does not directly prime the to-be-matched targets; then, cue ineffectiveness could mean that a change of the completion over mosaic balance of segmentation solutions is irrelevant, because they are both active, independent of their relative strength. On the contrary, suppose that the cue does not affect the segmentation of the composite prime; then, cue ineffectiveness runs counter a possible direct priming action by the cue on to-be-matched targets.

As regards prime duration, no effect was found. This result could follow from the substantial irrelevance of the difference between 50- and 500-ms exposures in the absence of backward masking. The structural simplicity of prime displays, combined with their repetition over the experimental session, made them equally available independent of exposure duration, obscuring the possible time course of amodal completion.

## Supplemental Material

Supplemental material for Mid-level Priming by Completion vs. Mosaic Solutions Click here for additional data file. Supplemental Material for Mid-level Priming by Completion vs. Mosaic Solutions by Antonio Peta, Carlo Fantoni and Walter Gerbino in i-Perception

## Figures and Tables

**Figure A.1.**
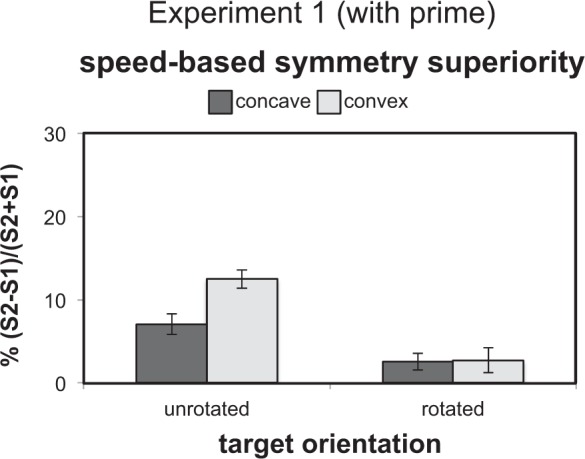
Experiment 1. Mean values (± 1 sem) of speed-based symmetry superiority in the reduced Rotation × CCP design. Symmetry superiority was larger than zero in each condition. The expected priming effect consisted in the large difference between symmetry superiorities for convex (hexagons over pentagons) vs. concave (hourglasses over pacmen) targets in the unrotated condition, unparalleled in the rotated condition.

**Figure A.2.**
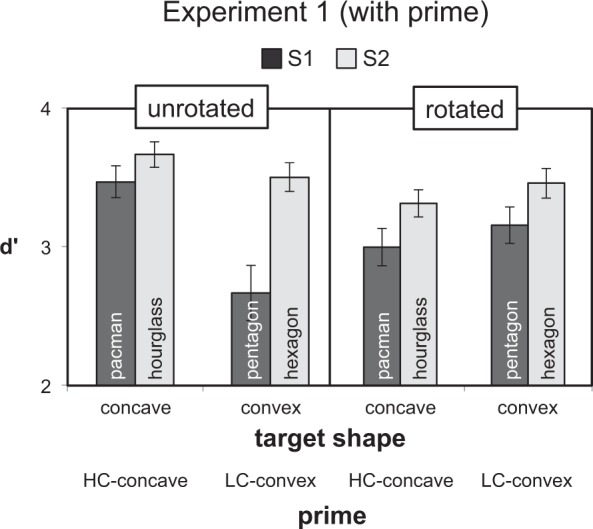
Experiment 1. Mean *d′* values (± 1 sem) in the reduced within-subjects Rotation × CCP × Symmetry design.

**Figure A.3.**
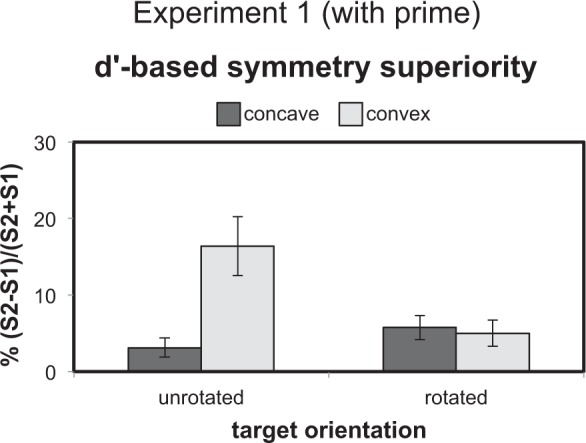
Experiment 1. Mean values (± 1 sem) of *d′*-based symmetry superiority in the reduced Rotation × CCP design. Symmetry superiority was larger than zero in each condition.

**Figure A.4.**
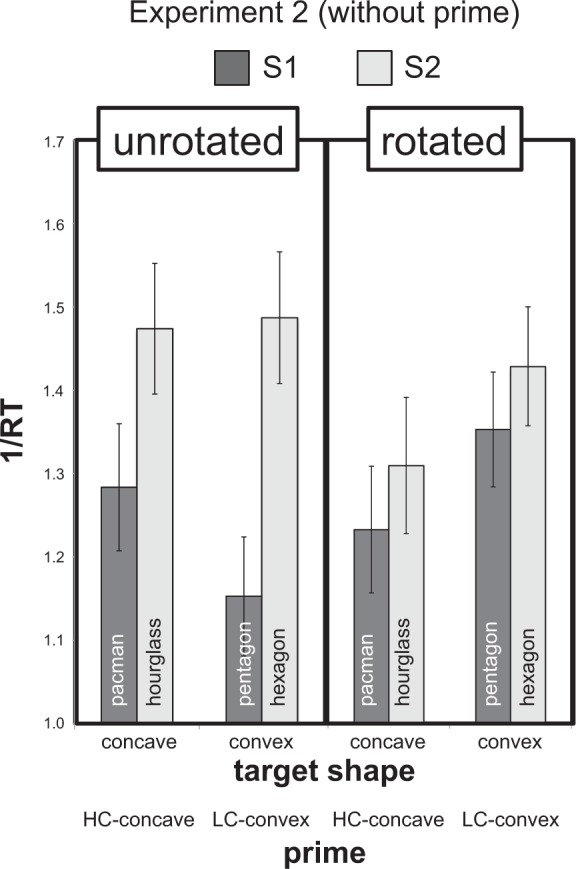
Experiment 2. Mean values of 1/RT (± 1 sem) in the within-subjects Rotation × CCP × Symmetry design.

**Figure A.5.**
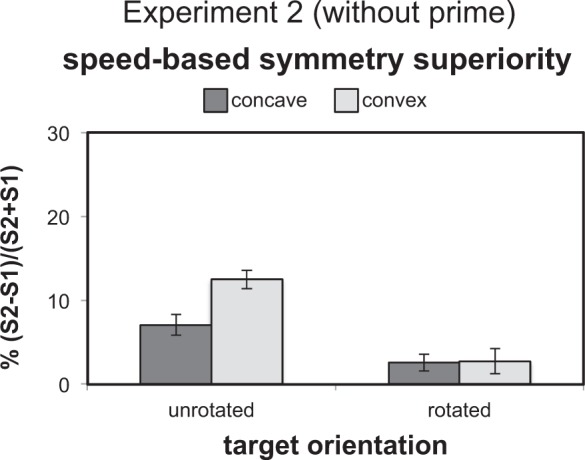
Experiment 2. Mean values (± 1 sem) of speed-based symmetry superiority in the reduced Rotation × CCP design.

**Figure A.6.**
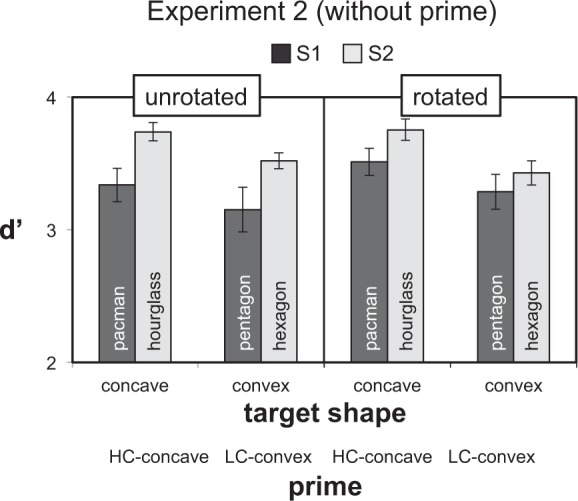
Experiment 2. Mean *d′* values in the Rotation × CCP × Symmetry design. The pattern was only partially similar to the one for response speed in Experiment 2, shown in Figure A.5.

**Figure A.7.**
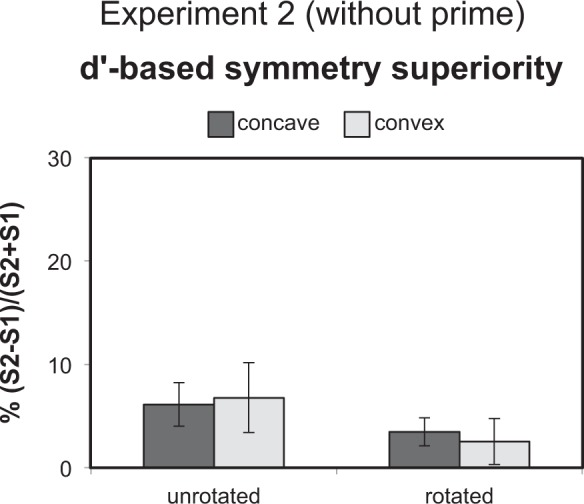
Experiment 2. Mean amounts of *d′*-based symmetry superiority in the reduced Rotation × CCP design.

## Author Note

Antonio Peta is also affiliated to Department of Psychology, University of Bologna, Italy.

## Notes

1 Earlier labels for the same concept are “representation without color” (Koffka, 1935, p. 178) and “invisibly present” (*unsichtbar vorhanden*, Metzger, 1936/2006, Chapter 8).

2 For knowledge-based completion priming, see Yun et al. (2018).

3 Cue: *F* < 1; Exposure: *F*(1, 2869.5) = 2.07, *p* = .150; Cue × Rotation: *F* < 1; Cue × CCP: *F* < 1; Cue ×Symmetry: *F*(1, 2872.2.5) = 1.61, *p* < .20; Cue × Exposure: *F* < 1; Exposure × Rotation: *F*(1, 2869.5) = 1.70, *p* = .193; Exposure × CCP: *F* < 1; Exposure × Symmetry: *F*(1, 2869.5) = 1.83, *p* = .176; Rotation × Symmetry × Cue: *F* < 1; Rotation × Cue × Exposure: *F*(1, 2869.5) = 1.489, *p* = .222; Rotation × CCP × Exposure: *F*(1, 2869.5) = 2.69, *p* = .101; Rotation × Symmetry × Exposure: *F* < 1.

4 CCP: *F*(1, 2872.3) = 29.05, *p* < .001, $\eta_{\text{p}}^{2}$ = 0.010; Rotation: *F*(1, 2869.6) = 3.44, *p* = .064; Rotation × CCP: *F*(1, 2869.7) = 44.14, *p* < .001, $\eta_{\text{p}}^{2}$ = 0.015; Rotation × Symmetry: *F*(1, 2869.6) = 6.09, *p* < .02, $\eta_{\text{p}}^{2}$ = 0.002; CCP × Symmetry: *F*(1, 2872.3) = 14.75, *p* < .001, $\eta_{\text{p}}^{2}$ = 0.005.

## Appendix

### Response speed in Experiment 1

We ran a 3-way repeated-measures ANOVA on the Rotation × CCP × Symmetry design, using the trimean of correct *same* response speeds as the individual performance score. The pattern of 8 group means was almost indistinguishable from the one in left graph of Figure 6 (therefore, we do not report it here) and the same was true for the significance of differences, with respect to the output of the LMER analysis of the 5-factor design. Apart from the main effect of Rotation [*F*(1, 24) = 2.24, *p* = 0.148], all other effects were significant [CCP: *F*(1, 24) = 23.50, *p* < 0.001, $\eta_{\text{p}}^{2}$ = 0.495; Symmetry: *F*(1, 24) = 54.30, *p* < 0.001, $\eta_{\text{p}}^{2}$ = 0.693; Rotation × CCP: *F*(1, 24) = 61.80, *p* < 0.001, $\eta_{\text{p}}^{2}$ = 0.720; Rotation × Symmetry: *F*(1, 24) = 7.06, *p*< 0.001, $\eta_{\text{p}}^{2}$ = 0.227; CCP × Symmetry: *F*(1, 24) = 7.68, *p* < 0.02, $\eta_{\text{p}}^{2}$ = 0.242; Rotation × CCP × Symmetry: *F*(1, 24)= 4.81, *p* < 0.05, $\eta_{\text{p}}^{2}$ = 0.167].

To better understand the determinants of response speed, we ran two separate ANOVAs for unrotated vs. rotated conditions, expecting a main effect of Symmetry in both conditions and a CCP × Symmetry interaction in the unrotated condition only. In the unrotated condition each main effect [CCP: *F*(1, 24) = 94.60, *p* < 0.001, $\eta_{\text{p}}^{2}$ = 0.798; Symmetry: *F*(1, 24) = 70.50, *p* < 0.001, $\eta_{\text{p}}^{2}$ = 0.746], as well as the CCP × Symmetry interaction [*F*(1,24) = 8.84, *p* < 0.01, $\eta_{\text{p}}^{2}$ = 0.269], were significant; while in the rotated condition only the main effect of Symmetry was significant [*F*(1, 24) = 23.30, *p* < 0.001, $\eta_{\text{p}}^{2}$ =0.493; CCP and 2-way interaction: *F <* 1].

The pattern of response speeds is summarized in Figure A.1, which displays the amount of symmetry superiority in the four conditions of the Rotation × CCP design. The symmetry superiority was larger than zero in each of the four conditions (*p* < 0.01). A 2-way ANOVA showed the significance of all effects [Rotation: *F*(1, 24) = 8.13, *p* < 0.01, $\eta_{\text{p}}^{2}$ = 0.253; CCP: *F*(1, 24) = 10.20, *p* < 0.005, $\eta_{\text{p}}^{2}$ = 0.298; Rotation × CCP interaction: *F*(1, 24) = 6.95, *p* < 0.02, $\eta_{\text{p}}^{2}$ = 0.225]. The mean symmetry superiority for unrotated convex targets (hexagons vs. pentagons) was larger than the mean symmetry superiority for unrotated concave targets (hourglasses vs. pacmen) [11.36 vs. 4.28%: *t*(24)= 3.486, *p* < 0.002, Hedges’s *g* = 0.945]. The two means in the rotated condition did not differ [4.10 vs. 5.49%: *t*(24)= 1.062, *p* = 0.299]. As apparent in Figure A.1, the planned contrast between *D*_u_ and *D*_r_ for response speed was significant [7.08 vs. 1.40%; *t*(24) = 2.64, *p* < 0.02, Hedges’s *g* = 0.634].

To evaluate the facilitatory and inhibitory components of the overall priming effect we compared mean speeds in unrotated vs. rotated conditions within each CCP level. The mean symmetry superiority for convex targets (hexagons vs. pentagons) was larger in the unrotated than rotated condition [11.36 vs. 5.49%: *t*(24)= 3.306, *p* < 0.005, Hedges’s *g* = 0.713], supporting the facilitatory component of priming. Mean symmetry superiorities for concave targets (hourglasses vs. pacmen) in unrotated vs. rotated conditions did not differ [4.29 vs. 4.10%: *t* < 1], against the possible inhibitory component of priming.

### d′ *in Experiment 1*

The same analysis was run on *d′* values. Recall that this parameter could not be computed for the full 5-factor design, where the number of repeated trials in each cell was too low to obtain reliable estimates of *p*(Hit) and *p*(FA). In the reduced Rotation × CCP × Symmetry design the lowest number of Hits was 1 out of 16 positive trials per cell, while the highest number of FAs was 9 out of 32 negative trials per cell. After the correction for extreme proportions, the 200 *d′* values (8 conditions × 25 participants) ranged from 0.620 to 4.017 (mean= 3.277; median= 3.397).

The pattern of mean *d′* values (Figure A.2) was remarkably similar to the one for response speed in Figure 6. The Rotation × CCP × Symmetry within-subjects ANOVA and subsequent CCP × Symmetry ANOVAs for unrotated vs. rotated conditions replicated the outcome of the same analyses on response speeds. With the exception of the main effect of Rotation [*F*(1, 24) = 1.54, *p* = 0.227], all other effects were significant in the 3-way ANOVA [CCP: *F*(1, 24) = 4.42, *p*< 0.05, $\eta_{\text{p}}^{2}$ = 0.156; Symmetry: *F*(1, 24) = 34.00, *p* < 0.001, $\eta_{\text{p}}^{2}$ = 0.586; Rotation × CCP: *F*(1, 24) = 13.90, *p* < 0.002, $\eta_{\text{p}}^{2}$ = 0.367; Rotation × Symmetry: *F*(1, 24) = 4.59, *p* < 0.05, $\eta_{\text{p}}^{2}$ = 0.161; CCP × Symmetry: *F*(1, 24) = 6.58, *p* < 0.02, $\eta_{\text{p}}^{2}$ = 0.215; Rotation × CCP × Symmetry: *F*(1, 24) = 8.78, *p* < 0.01, $\eta_{\text{p}}^{2}$ = 0.268]. In the unrotated condition both the main effects [CCP: *F*(1, 24) = 16.70, *p* < 0.001, $\eta_{\text{p}}^{2}$ = 0.410; Symmetry: *F*(1, 24) = 27.50, *p* < 0.001, $\eta_{\text{p}}^{2}$ = 0.534] and the CCP × Symmetry interaction [*F*(1,24) = 11.40, *p* < 0.01, $\eta_{\text{p}}^{2}$ = 0.322] were significant; while in the rotated condition only the main effect of Symmetry was significant [*F*(1, 24) = 21.10, *p* < 0.001, $\eta_{\text{p}}^{2}$ = 0.468; CCP: *F*(1, 24) = 1.81, *p* = 0.191; CCP × Symmetry: *F <* 1].

Figure A.3 displays the amount of *d′*-based symmetry superiority in the four conditions of the Rotation × CCP design. Symmetry superiority was larger than zero in each of the four conditions (*p*< 0.02 in the unrotated-concave conditions; *p*< 0.01 in the other three conditions). A 2-way ANOVA showed the significance of all effects [Rotation: *F*(1, 24) = 5.53, *p* < 0.05, $\eta_{\text{p}}^{2}$ = 0.187; CCP: *F*(1, 24) = 7.25, *p* < 0.02, $\eta_{\text{p}}^{2}$ = 0.232; Rotation × CCP interaction: *F*(1, 24) = 10.40, *p* < 0.005, $\eta_{\text{p}}^{2}$ = 0.302]. The mean symmetry superiority for unrotated convex targets (hexagons vs. pentagons) was larger than the mean symmetry superiority for unrotated concave targets (hourglasses vs. pacmen) [16.40 vs. 3.13%: *t*(24)= 3.437, *p* < 0.005, Hedges’s *g* = 0.879]. The two means in the rotated condition did not differ [5.76 vs. 5.00%: *t* < 1]. As apparent in Figure A.3, the planned contrast between *d′*-based *D*_u_ and *D*_r_ was also significant [*D*_u_ = 13.27% vs. *D*_r_ = -0.76%; *t*(24) = 3.22, *p* < 0.005, Hedges’s *g* = 0.848].

To evaluate the facilitatory and inhibitory components of the overall priming effect we compared *d′* values in unrotated vs. rotated conditions within each CCP level. The *d′*-based mean symmetry superiority for convex targets (hexagon vs. pentagon) was larger in the unrotated than rotated condition [16.40 vs. 5.00%: t(24)= 3.160, p < 0.005, Hedges’s *g* = 0.691]. Mean symmetry superiorities for concave targets (hourglasses vs. pacmen) in unrotated vs. rotated conditions did not differ [3.13 vs. 5.76%: t(24)= 1.43, *p* = 0.166], against the possible inhibitory component of priming.

### Response speed in Experiment 2

Figure A.4 shows mean response speeds in the 8 conditions of the Rotation × CCP × Symmetry design of Experiment 2. The main effect of Symmetry was significant [*F*(1, 19) = 95.40, *p* < 0.001, $\eta_{\text{p}}^{2}$ = 0.834], while the other two main effects were not [Rotation: *F*(1, 19) = 3.47, *p* = 0.078; CCP: *F*(1, 19) = 1.23, *p* = 0.281]. All 2-way interaction were significant [Rotation × CCP: *F*(1, 19) = 51.90, *p* < 0.001, $\eta_{\text{p}}^{2}$ = 0.732; Rotation × Symmetry: *F*(1, 19) = 59.40, *p* < 0.001, $\eta_{\text{p}}^{2}$ = 0.758; CCP × Symmetry: *F*(1, 19) = 6.72, *p* < 0.02, $\eta_{\text{p}}^{2}$ = 0.261], while the Rotation × CCP × Symmetry interaction did not reach significance [*F*(1, 19) = 3.15, *p* = 0.092].

Then, we evaluated the speed-based symmetry superiority in the Rotation × CCP design (Figure A.5). The outcome of the 2-way ANOVA differed from the one for Experiment 1, despite some similarities between the two patterns of means (see Figure A.1 for comparison). Both main effects were significant [Rotation: *F*(1, 19) = 53.80, *p* < 0.001, $\eta_{\text{p}}^{2}$ = 0.739; CCP: *F*(1, 19) = 6.24, *p* < 0.05, $\eta_{\text{p}}^{2}$ = 0.230], but the Rotation × CCP interaction was not [*F*(1, 19) = 3.62, *p* = 0.072], paralleling the lack of significance of the Rotation × CCP × Symmetry interaction on response speed.

### d′ *in Experiment 2*

Differently from Experiment 1, in Experiment 2 the pattern of mean *d′* values (Figure A.6) did not parallel the one for response speed (Figure A.4). The 3-way ANOVA on *d′* values showed the significance of the main effects of CCP [*F*(1, 19) = 17.10, *p* < 0.001, $\eta_{\text{p}}^{2}$ = 0.474] and Symmetry [*F*(1, 19) = 6.61, *p* < 0.02, $\eta_{\text{p}}^{2}$ = 0.258], and of the Rotation × Symmetry interaction [*F*(1,19) = 5.16, *p* < 0.05, $\eta_{\text{p}}^{2}$ = 0.214], while the main effect of Rotation [*F*(1,19) = 1.48, *p* < 0.238], the other 2-way interactions [*F* < 1] and the 3-way interaction [*F* < 1] were not.

In Experiment 2 the pattern of *d′*-based symmetry superiorities in the Rotation × CCP design (Figure A.7) differed from the one for response speed (Figure A.5). Only the main effect of Rotation was significant [*F*(1,19) = 5.40, *p* < 0.05, $\eta_{\text{p}}^{2}$ = 0.221], while the main effect of CCP and the 2-way interaction were not [*F* < 1].
