# Supplementary material for: Mid-level Priming by Completion vs. Mosaic Solutions
Source: Iperception. 2019 Apr 29;10(2):2041669518820347. doi: 10.1177/2041669518820347 (PMC6492368; doi:10.1177/2041669518820347)
Supplement: Supplemental material for Mid-level Priming by Completion vs. Mosaic Solutions [file Supplemental_Material.pdf]

Appendix

Response speed in Experiment 1

We ran a 3-way repeated-measures ANOVA on the Rotation × CCP × Symmetry design, using the trimean of correct *same* response speeds as the individual performance score. The pattern of 8 group means was almost indistinguishable from the one in left graph of Figure 6 (therefore, we do not report it here) and the same was true for the significance of differences, with respect to the output of the LMER analysis of the 5-factor design. Apart from the main effect of Rotation [$F(1, 24) = 2.24$, $p = 0.148$], all other effects were significant [CCP: $F(1, 24) = 23.50$, $p < 0.001$, $\eta^2_p = 0.495$; Symmetry: $F(1, 24) = 54.30$, $p < 0.001$, $\eta^2_p = 0.693$; Rotation × CCP: $F(1, 24) = 61.80$, $p < 0.001$, $\eta^2_p = 0.720$; Rotation × Symmetry: $F(1, 24) = 7.06$, $p < 0.001$, $\eta^2_p = 0.227$; CCP × Symmetry: $F(1, 24) = 7.68$, $p < 0.02$, $\eta^2_p = 0.242$; Rotation × CCP × Symmetry: $F(1, 24) = 4.81$, $p < 0.05$, $\eta^2_p = 0.167$].

To better understand the determinants of response speed, we ran two separate ANOVAs for unrotated vs. rotated conditions, expecting a main effect of Symmetry in both conditions and a CCP × Symmetry interaction in the unrotated condition only. In the unrotated condition each main effect [CCP: $F(1, 24) = 94.60$, $p < 0.001$, $\eta^2_p = 0.798$; Symmetry: $F(1, 24) = 70.50$, $p < 0.001$, $\eta^2_p = 0.746$], as well as the CCP × Symmetry interaction [$F(1,24) = 8.84$, $p < 0.01$, $\eta^2_p = 0.269$], were significant; while in the rotated condition only the main effect of Symmetry was significant [$F(1, 24) = 23.30$, $p < 0.001$, $\eta^2_p = 0.493$; CCP and 2-way interaction: $F < 1$].

The pattern of response speeds is summarized in Figure A.1, which displays the amount of symmetry superiority in the four conditions of the Rotation × CCP design. The symmetry superiority was larger than zero in each of the four conditions ($p < 0.01$). A 2-

way ANOVA showed the significance of all effects [Rotation: $F(1, 24) = 8.13$, $p < 0.01$, $\eta^2_p = 0.253$; CCP: $F(1, 24) = 10.20$, $p < 0.005$, $\eta^2_p = 0.298$; Rotation × CCP interaction: $F(1, 24) = 6.95$, $p < 0.02$, $\eta^2_p = 0.225$]. The mean symmetry superiority for unrotated convex targets (hexagons vs. pentagons) was larger than the mean symmetry superiority for unrotated concave targets (hourglasses vs. pacmen) [11.36 vs. 4.28%: $t(24)= 3.486$, $p < 0.002$, Hedges's $g = 0.945$]. The two means in the rotated condition did not differ [4.10 vs. 5.49%: $t(24)= 1.062$, $p = 0.299$]. As apparent in Figure A.1, the planned contrast between D_u and D_r for response speed was significant [7.08 vs. 1.40%; $t(24) = 2.64$, $p < 0.02$, Hedges's $g = 0.634$].

To evaluate the facilitatory and inhibitory components of the overall priming effect we compared mean speeds in unrotated vs. rotated conditions within each CCP level. The mean symmetry superiority for convex targets (hexagons vs. pentagons) was larger in the unrotated than rotated condition [11.36 vs. 5.49%: $t(24)= 3.306$, $p < 0.005$, Hedges's $g = 0.713$], supporting the facilitatory component of priming. Mean symmetry superiorities for concave targets (hourglasses vs. pacmen) in unrotated vs. rotated conditions did not differ [4.29 vs. 4.10%: $t < 1$], against the possible inhibitory component of priming.

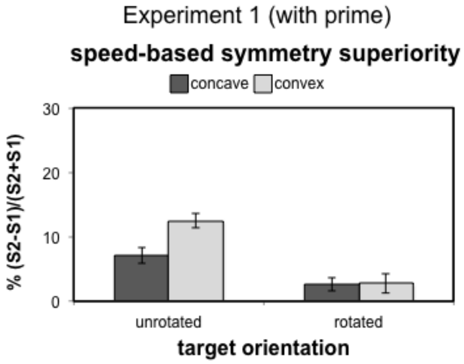

Figure A.1. Experiment 1. Mean values (± 1 sem) of speed-based symmetry superiority in the reduced Rotation × CCP design. Symmetry superiority was larger than zero in each condition. The expected priming effect consisted in the large difference between symmetry superiorities for convex (hexagons over pentagons) vs. concave (hourglasses over pacmen) targets in the unrotated condition, ~~unparalled~~ in the rotated condition.

d' *in Experiment 1*

The same analysis was ~~ran~~ on *d'* values. Recall that this parameter could not be computed for the full 5-factor design, where the number of repeated trials in each cell was too low to obtain reliable estimates of *p*(Hit) and *p*(FA). In the reduced Rotation × CCP × Symmetry design the lowest number of Hits was 1 out of 16 positive trials per cell, while the highest number of FAs was 9 out of 32 negative trials per cell. After the correction for extreme proportions, the 200 *d'* values (8 conditions × 25 participants) ranged from 0.620 to 4.017 (mean= 3.277; median= 3.397).

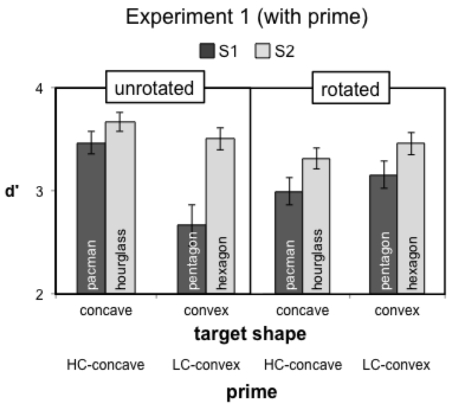

Figure A.2. Experiment 1. Mean *d'* values (± 1 sem) in the reduced within-subjects Rotation × CCP × Symmetry.

The pattern of mean *d'* values (Figure A.2) was remarkably similar to the one for response speed in Figure 6. The Rotation × CCP × Symmetry within-subjects ANOVA and subsequent CCP × Symmetry ANOVAs for unrotated vs. rotated conditions replicated the outcome of the same analyses on response speeds. With the exception of the main effect of Rotation [$F(1, 24) = 1.54$, $p = 0.227$], all other effects were significant in the 3-way ANOVA [CCP: $F(1, 24) = 4.42$, $p < 0.05$, $\eta^2_p = 0.156$; Symmetry: $F(1, 24) = 34.00$, $p < 0.001$, $\eta^2_p = 0.586$; Rotation × CCP: $F(1, 24) = 13.90$, $p < 0.002$, $\eta^2_p = 0.367$; Rotation × Symmetry: $F(1, 24) = 4.59$, $p < 0.05$, $\eta^2_p = 0.161$; CCP × Symmetry: $F(1, 24) = 6.58$, $p < 0.02$, $\eta^2_p = 0.215$; Rotation × CCP × Symmetry: $F(1, 24) = 8.78$, $p < 0.01$, $\eta^2_p = 0.268$]. In the unrotated condition both the main effects [CCP: $F(1, 24) = 16.70$, $p < 0.001$, $\eta^2_p = 0.410$; Symmetry: $F(1, 24) = 27.50$, $p < 0.001$, $\eta^2_p = 0.534$] and the CCP × Symmetry interaction [$F(1,24) = 11.40$, $p < 0.01$, $\eta^2_p = 0.322$] were significant; while in the rotated condition only the main effect of Symmetry was significant [$F(1, 24) = 21.10$, $p < 0.001$, $\eta^2_p = 0.468$; CCP: $F(1, 24) = 1.81$, $p = 0.191$; CCP × Symmetry: $F < 1$].

Figure A.3 displays the amount of *d'*-based symmetry superiority in the four conditions of the Rotation × CCP design. Symmetry superiority was larger than zero in each of the four conditions ($p < 0.02$ in the unrotated-concave conditions; $p < 0.01$ in the other three conditions). A 2-way ANOVA showed the significance of all effects [Rotation: $F(1, 24) = 5.53$, $p < 0.05$, $\eta^2_p = 0.187$; CCP: $F(1, 24) = 7.25$, $p < 0.02$, $\eta^2_p = 0.232$; Rotation × CCP interaction: $F(1, 24) = 10.40$, $p < 0.005$, $\eta^2_p = 0.302$]. The mean symmetry superiority for unrotated convex targets (hexagons vs. pentagons) was larger than the mean symmetry superiority for unrotated concave targets (hourglasses vs.

pacmen) [16.40 vs. 3.13%: $t(24) = 3.437$, $p < 0.005$, Hedges's $g = 0.879$]. The two means in the rotated condition did not differ [5.76 vs. 5.00%: $t < 1$]. As apparent in Figure A.3, the planned contrast between *d'*-based D_u and D_r was also significant [D_u = 13.27% vs. D_r = -0.76%; $t(24) = 3.22$, $p < 0.005$, Hedges's $g = 0.848$].

To evaluate the facilitatory and inhibitory components of the overall priming effect we compared *d'* values in unrotated vs. rotated conditions within each CCP level. The *d'*-based mean symmetry superiority for convex targets (hexagon vs. pentagon) was larger in the unrotated than rotated condition [16.40 vs. 5.00%: $t(24) = 3.160$, $p < 0.005$, Hedges's $g = 0.691$]. Mean symmetry superiorities for concave targets (hourglasses vs. pacmen) in unrotated vs. rotated conditions did not differ [3.13 vs. 5.76%: $t(24) = 1.43$, $p = 0.166$], against the possible inhibitory component of priming.

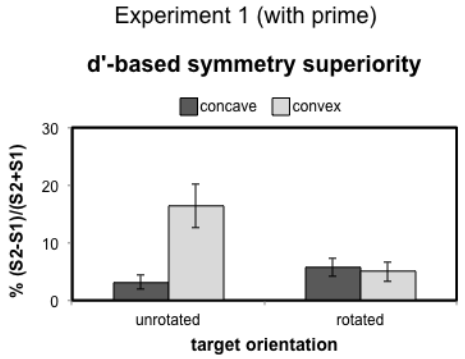

Figure A.3. Experiment 1. Mean values (± 1 sem) of *d'*-based symmetry superiority in the reduced Rotation × CCP design. Symmetry superiority was larger than zero in each condition.

Response speed in Experiment 2

Figure A.4 shows mean response speeds in the 8 conditions of the Rotation × CCP × Symmetry design of Experiment 2. The main effect of Symmetry was significant [$F(1, 19) = 95.40$, $p < 0.001$, $\eta^2_p = 0.834$], while the other two main effects were not [Rotation: $F(1, 19) = 3.47$, $p = 0.078$; CCP: $F(1, 19) = 1.23$, $p = 0.281$]. All 2-way interaction were

significant [Rotation × CCP: $F(1, 19) = 51.90$, $p < 0.001$, $\eta^2_p = 0.732$; Rotation × Symmetry: $F(1, 19) = 59.40$, $p < 0.001$, $\eta^2_p = 0.758$; CCP × Symmetry: $F(1, 19) = 6.72$, $p < 0.02$, $\eta^2_p = 0.261$], while the Rotation × CCP × Symmetry interaction did not reach significance [$F(1, 19) = 3.15$, $p = 0.092$].

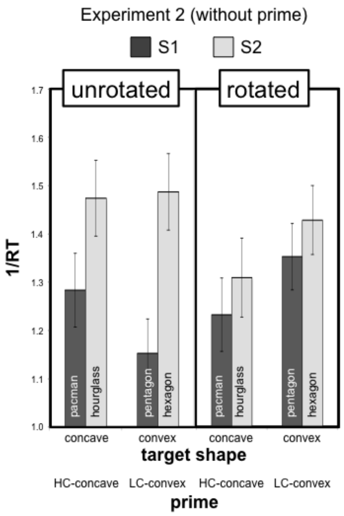

Figure A.4. Experiment 2. Mean values of 1/RT (± 1 sem) in the within-subjects Rotation × CCP × Symmetry design.

Then, we evaluated the speed-based symmetry superiority in the Rotation × CCP design (Figure A.5). The outcome of the 2-way ANOVA differed from the one for Experiment 1, despite some similarities between the two pattern of means (see Figure A.1 for comparison). Both main effects were significant [Rotation: $F(1, 19) = 53.80$, $p < 0.001$, $\eta^2_p = 0.739$; CCP: $F(1, 19) = 6.24$, $p < 0.05$, $\eta^2_p = 0.230$], but the Rotation × CCP interaction was not [$F(1, 19) = 3.62$, $p = 0.072$], paralleling the lack of significance of the Rotation × CCP × Symmetry interaction on response speed.

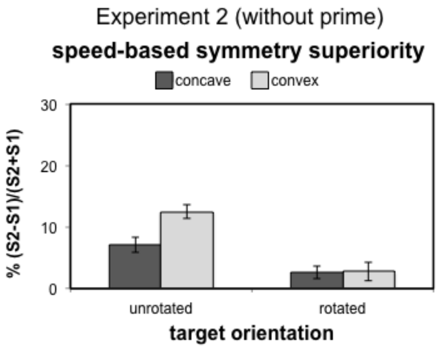

Figure A.5. Experiment 2. Mean values (± 1 sem) of speed-based symmetry superiority in the reduced Rotation × CCP design.

d’ *in Experiment 2*

Differently from Experiment 1, in Experiment 2 the pattern of mean *d’* values (Figure A.6) did not parallel the one for response speed (Figure A.4). The 3-way ANOVA on *d’* values showed the significance of the main effects of CCP [$F(1, 19) = 17.10$, $p < 0.001$, $\eta^2_p = 0.474$] and Symmetry [$F(1, 19) = 6.61$, $p < 0.02$, $\eta^2_p = 0.258$], and of the Rotation × Symmetry interaction [$F(1,19) = 5.16$, $p < 0.05$, $\eta^2_p = 0.214$], while the main effect of Rotation [$F(1,19) = 1.48$, $p < 0.238$], the other 2-way interactions [$F < 1$] and the 3-way interaction [$F < 1$] were not.

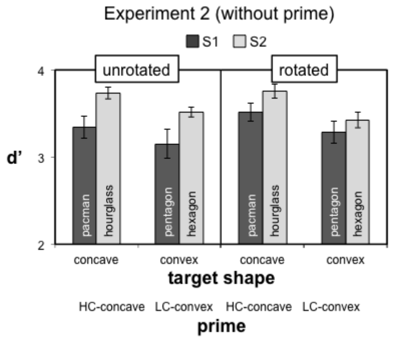

Figure A.6. Experiment 2. Mean *d’* values in the Rotation × CCP × Symmetry design. The pattern was only partially similar to the one for response speed in Experiment 2, shown in Figure A.5.

In Experiment 2 the pattern of *d'*-based symmetry superiorities in the Rotation × CCP design (Figure A.7) differed from the one for response speed (Figure A.5). Only the main effect of Rotation was significant [$F(1,19) = 5.40$, $p < 0.05$, $\eta^2_p = 0.221$], while the main effect of CCP and the 2-way interaction were not [$F < 1$].

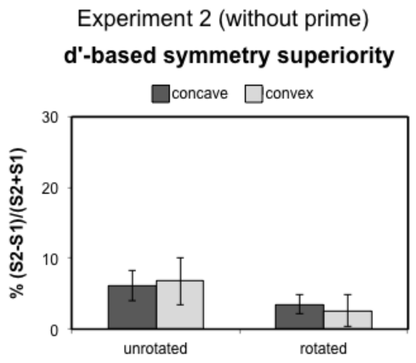

Figure A.7. Experiment 2. Mean amounts of *d'*-based symmetry superiority in the reduced Rotation × CCP design.
